# A novel serum-free medium for the expansion of human mesenchymal stem cells

**DOI:** 10.1186/scrt8

**Published:** 2010-04-02

**Authors:** Lucas G Chase, Uma Lakshmipathy, Luis A Solchaga, Mahendra S Rao, Mohan C Vemuri

**Affiliations:** 1 Primary and Stem Cell Systems, Life Technologies, 501 Charmany Drive, Madison, WI 53719, USA; 2 Primary and Stem Cell Systems, 5781 Van Allen Way, Carlsbad, CA 92008, USA; 3 Department of General Medical Sciences, Division of Hematology and Oncology, Case Western Reserve University, 2103 Cornell Road, Cleveland, OH 44106, USA; 4 Primary and Stem Cell Systems, Life Technologies, 7335 Executive Way, Frederick, MD 21704, USA

## Abstract

**Introduction:**

Human multipotent mesenchymal stem cell (MSC) therapies are being tested clinically for a variety of disorders, including Crohn's disease, multiple sclerosis, graft-*versus*-host disease, type 1 diabetes, bone fractures, and cartilage defects. However, despite the remarkable clinical advancements in this field, most applications still use traditional culture media containing fetal bovine serum. The ill-defined and highly variable nature of traditional culture media remains a challenge, hampering both the basic and clinical human MSC research fields. To date, no reliable serum-free medium for human MSCs has been available.

**Methods:**

In this study, we developed and tested a serum-free growth medium on human bone marrow-derived MSCs through the investigation of multiple parameters including primary cell isolation, multipassage expansion, mesoderm differentiation, cellular phenotype, and gene-expression analysis.

**Results:**

Similar to that achieved with traditional culture medium, human MSCs expanded in serum-free medium supplemented with recombinant human platelet-derived growth factor-BB (PDGF-BB), basic fibroblast growth factor (bFGF), and transforming growth factor (TGF)-β1 showed extensive propagation with retained phenotypic, differentiation, and colony-forming unit potential. To monitor global gene expression, the transcriptomes of bone marrow-derived MSCs expanded under serum-free and serum-containing conditions were compared, revealing similar expression profiles. In addition, the described serum-free culture medium supported the isolation of human MSCs from primary human marrow aspirate with continual propagation.

**Conclusions:**

Although the described serum-free MSC culture medium is not free of xenogeneic components, this medium provides a substitute for serum-containing medium for research applications, setting the stage for future clinical applications.

## Introduction

The human bone marrow contains a distinct stromal cell fraction referred to as multipotent mesenchymal stem (stromal) cells or MSCs [1]. Although the stromal fraction of bone marrow was originally considered to be merely a structural supportive framework for the hematopoietic system, numerous studies have now shown that MSCs can give rise to a wide array of mesenchymal cell types, including bone, fat, and cartilage [1]. Since the first published report by Friedenstein and colleagues [2] describing the expansion of an adherent, spindle-shaped population of cells from whole human bone marrow, MSC or MSC-like cells have also been expanded from numerous other compartments, including skeletal muscle, adipose tissue, umbilical cord, synovium, dental pulp, amniotic fluid, human embryonic stem cells, and numerous other sources [3,4]. Although much focus has been placed on the use of MSCs for cell-based therapies [5], more recently, a great deal of attention has been given to the use of MSCs for paracrine support and immune modulation, including the prevention of graft-*versus*-host disease [6-8].

As it has been estimated that human MSCs comprise a mere 0.001 to 0.01% of total bone marrow mononuclear cells [9], this population requires extensive *in vitro *cell-culture expansion to obtain sufficient numbers for basic biologic or clinical applications. Historically, MSC culture medium has comprised a basal culture medium (i.e. Dulbecco's Modified Eagle's Medium or Minimum Essential Medium alpha) supplemented with fetal bovine serum (FBS) with or without additional growth factors (i.e. bFGF). Although these traditional formulations provide robust undifferentiated MSC expansion, the ill-defined nature of FBS is undesirable for downstream research and therapeutic applications and provides inconsistent lot-to-lot performance. To overcome the inconsistent performance associated with FBS, commercial vendors and researchers alike have implemented quality-control measures in which individual lots of FBS are prescreened for performance, a costly and time-consuming activity. In addition, the variability associated with FBS results in an overall inconsistent reagent and thereby introduces variability between experimental results, making data comparisons (i.e. cellular differentiation, genomics, and proteomics) more difficult. To circumvent this issue, a robust serum-free MSC culture medium has become absolutely necessary. We describe here a more-defined, serum-free MSC culture medium that can support the robust expansion of undifferentiated human MSCs. Characterization of this serum-free expanded MSC population reveals a similar phenotype and expression profile compared with cells expanded in traditional serum-containing medium, while maintaining the defining growth and differentiation characteristics of human MSCs. In addition, it is revealed that PDGF-BB, bFGF, and TGF-β1, three growth factors we have previously shown to support the expansion of undifferentiated human MSCs [10], support human MSC expansion in a synergistic manner in this serum-free formulation. Although the described serum-free MSC culture medium is not free of xenogeneic components, this work sets the stage for serum-free MSC cell culture and thereby provides a necessary research tool for the basic biologic understanding of human MSCs cultured under serum-free conditions.

## Materials and methods

### Human bone marrow MSC donor pool generation

To generate a passage 4, four-donor pool, human MSCs were obtained from two sources: (a) cryopreserved human MSCs or (b) human MSCs isolated from primary unprocessed human marrow (both from Lonza, Walkersville, MD). From each condition, MSCs were expanded in a serum-containing growth medium (SCM) consisting of low-glucose DMEM supplemented with 10% MSC-Qualified Fetal Bovine Serum (FBS), 2 mmol/L L-glutamine, and 5 μg/ml gentamicin (all from Invitrogen, Carlsbad, CA) to passage 4, where equal numbers of MSCs from each donor were combined into a single MSC donor pool and cryopreserved at 1.0 × 10^6 ^cells/ml (0.5 ml/vial) in complete SCM supplemented with 10% DMSO (Sigma-Aldrich, St. Louis, MO).

### Human bone marrow-derived MSC recovery and expansion

To recover pooled passage 4 human MSCs, each vial (5 × 10^5 ^cells) was retrieved from liquid nitrogen and was quickly thawed and added to a 50-ml conical tube. To the recovered cell suspension, 10 ml of SCM was added by drops (~5 drops/10 seconds with mixing) and placed directly into T225 culture flasks (BD Biosciences, Franklin Lakes, NJ) containing 30 ml of equilibrated SCM. Assuming ~ 90% cell recovery, the estimated postthaw seed density was ~2 × 10^3 ^cells/cm^2^. After 24 hours, complete culture medium was changed to remove residual DMSO and thereafter changed every 2 to 3 days until 70-90% confluence was attained. To test the ability of serum-free medium (SFM; StemPro^® ^MSC SFM, Invitrogen) or SCM to support the expansion of human MSCs, recovered four-donor pooled human MSCs (passage 5 total) were harvested by using TrypLE™ Express (Invitrogen) and seeded at 3,000 cells/cm^2 ^(SCM) or 10,000 cells/cm^2 ^(SFM) in triplicate wells of six-well plates (BD Biosciences). Throughout the duration of the cultures, individual wells remained separate and were propagated individually. For SFM conditions, culture vessels were coated with the defined, xenogeneic-free substrate CELLstart™ (1:100 dilution) or human plasma fibronectin (5 μg/cm^2^) (both from Invitrogen). On reaching ~70-90% confluence, all human MSC cultures were harvested by using TrypLE™ Express (Invitrogen), counted by using a Coulter Counter (Beckman-Coulter, Fullerton, CA), and reseeded at the same seeding density through the duration of the study.

### Primary human bone marrow-derived MSC isolation

Fresh unprocessed human bone marrow aspirate (Lonza) was obtained, and purified mononuclear cells were obtained by using a Histopaque-1077 density gradient (Sigma). To study the support of primary MSC expansion, 2.0 × 10^7 ^mononuclear cells were seeded into a T75 culture flask coated with 5 μg/cm^2 ^human plasma fibronectin (Invitrogen). After 3 days, complete medium was changed, and thereafter, changed every 2 to 3 days. After 8 days in culture, cells were harvested and propagated as described earlier.

### Cell-proliferation assay

To optimize growth-factor requirements in SFM, cryopreserved four-donor pooled human MSCs recovered in SCM were harvested and seeded into 96-well black-wall tissue culture-treated plates (Corning Incorporated, Corning, NY) at 3,000 cells/cm^2 ^in SCM or growth factor-deficient SFM supplemented with various combinations of the recombinant human growth factors PDGF-BB, bFGF, and TGF-β1 (all from Invitrogen). After 4 days in culture, cell proliferation was measured by using the CyQUANT^® ^NF Cell Proliferation Assay (Invitrogen) by using a Gemini EM microplate spectrofluorometer (Molecular Devices, Sunnyvale, CA).

### MSC differentiation and staining assays

Recovered passage 4 bone marrow MSCs from the four-donor pool (passage 5 total) were expanded in SFM (eight passages; passage 13 in total) and seeded into StemPro^® ^Osteogenesis, Adipogenesis or Chondrogenesis Differentiation medium (Invitrogen) in 12-well plates as per the manufacturers protocol. During differentiation, complete medium changes were made every 3 to 4 days. After 14 days, cultures were monitored for differentiation by using lineage-specific biologic stains. To monitor adipogenic differentiation, cultures were stained with Oil Red O (Sigma) (0.5% Oil Red O solution in 60% isopropanol), washed with distilled water, and visualized. For chondrogenic differentiation, micromass pellet cultures were fixed with 4% formaldehyde and stained with 1% Alcian Blue (Sigma). After staining, cultures were washed with 0.1N HCl and visualized. For osteogenesis, staining was performed by using the Leukocyte Alkaline Phosphatase Kit (Sigma), as per the manufacturer's protocol. In addition, to perform histologic staining on sectioned micromass pellet cultures, single-donor passage 1 bone marrow MSCs (isolated under standard SCM conditions) were propagated for three additional passages in SCM or SFM and were resuspended at 1.25 × 10^6 ^cells/ml in chondrogenic medium composed of high-glucose DMEM (Invitrogen) supplemented with 1% ITS+Premix (BD Biosciences), 100 μmol/L ascorbate-2-phosphate (WAKO, Richmond, VA), 10^-7 ^mol/L dexamethasone (Sigma), and 10 ng/ml TGFβ1 (Peprotech, Rocky Hill, NJ). To create chondrogenic micromass pellets, 2.5 × 10^5 ^cells from this cell solution were placed in 96-well polypropylene plates (Phenix, Hayward, CA), centrifuged at 500 × *g*, and placed in an incubator at 37°C, 95% air, and 5% CO_2_. Medium changes were made 3 times per week, and on day 21 of differentiation, cell pellets were harvested, fixed with 10% neutral-buffered formalin, paraffin-embedded, sectioned, and processed for histologic evaluation by staining with Toluidine blue.

### Flow cytometry

Passage 3 human MSCs expanded in SCM or SFM were harvested by using TrypLE™ Express and washed with DPBS (without Ca^2+^/Mg^2+^) supplemented with 5% FBS and stained with the following antibodies: CD11b (unconjugated, clone VIM12), CD34-APC (clone 581), CD45-AF405 (clone HI30), CD44-PE (clone MEM-85), CD105-APC (clone SN6), (all from Invitrogen); CD90 (clone 5E10), CD73-PE (clone AD2), (both from BD Biosciences); and where unconjugated primary antibodies were used, AlexaFluor^® ^488 anti-mouse (Invitrogen). To set background fluorescence levels, unstained and/or matched isotype controls were used.

### Colony-forming unit-fibroblast assay

MSC cultures (SFM and SCM) were harvested by using TrypLE™ Express, and the total cell number was determined by using a Coulter Counter. Harvested cells were reseeded into 5 × 100-mm tissue culture dishes (BD Biosciences) at 150 cells/dish (in SCM without subsequent medium changes). After 14 days, cultures were washed with DPBS and stained with 0.5% Crystal Violet (Sigma) in methanol for 5 to 10 minutes at room temperature. After staining, dishes were again washed with DPBS, allowed to dry, and colonies (those greater than 50 cells) were counted by using a dissecting microscope. Data were reported as total colony number per dish (per 150 cells). Standard deviation (SD) was calculated.

### RNA Isolation and qRT-PCR

To measure gene expression of selected surface antigens or other MSC markers, passage 4 human MSC cell pellets (single biologic replicate, triplicate technical replicates) were collected, and RNA was isolated by using the PureLink Micro-to-Midi Total RNA Purification Kit, and total RNA was DNase I-treated off-the-column, as per manufacturer's protocol. To generate cDNA and run real-time quantitative PCR (qRT-PCR), the SuperScript III Platinum SYBR Green Two-Step qRT-PCR Kit was used. qRT-PCR analysis was completed by using technical duplicates. The following primer sets were used:

CD73 - F:CGCAACAATGGCACAATTAC, R:CAGGTTTTCGGGAAAGATCA;

CD90 - F:TCGCTCTCCTGCTAACAGTCT, R:CTCGTACTGGATGGGTGAACT;

CD105 - F:CACTAGCCAGGTCTCGAAGG, R:CTGAGGACCAGAAGCACCTC;

NESTIN - F:ACCTCAAGATGTCCCTCAGC, R:TGGGAGCAAAGATCCAAGAC (all reagents for RNA isolation and qRT-PCR were from Invitrogen).

### Illumina microarray gene-expression analysis

Genome-wide expression profiles of human bone marrow-derived MSCs grown in SFM and SCM were obtained by using Illumina Bead Arrays. Total RNA was extracted using TRIzol and further purified of contaminating genomic DNA by using DNA-free (Ambion/Applied Biosystems, Foster City, CA) for 30 minutes at 37°C. RNA was precipitated and quantified spectrophotometrically, and its purity was assessed by gel electrophoresis. Sample amplification and biotinylated cRNA was prepared and generated from total RNA by using the Illumina total Prep kit (Ambion) according to the manufacturer's protocol. Array hybridization to the Sentrix Chip Array (Human WG-6) and processing procedures were performed according to protocols provided by the manufacturer (Illumina, San Diego, CA). Array data were processed and analyzed by using Illumina BeadStudio software.

## Results

**Figure 1:**
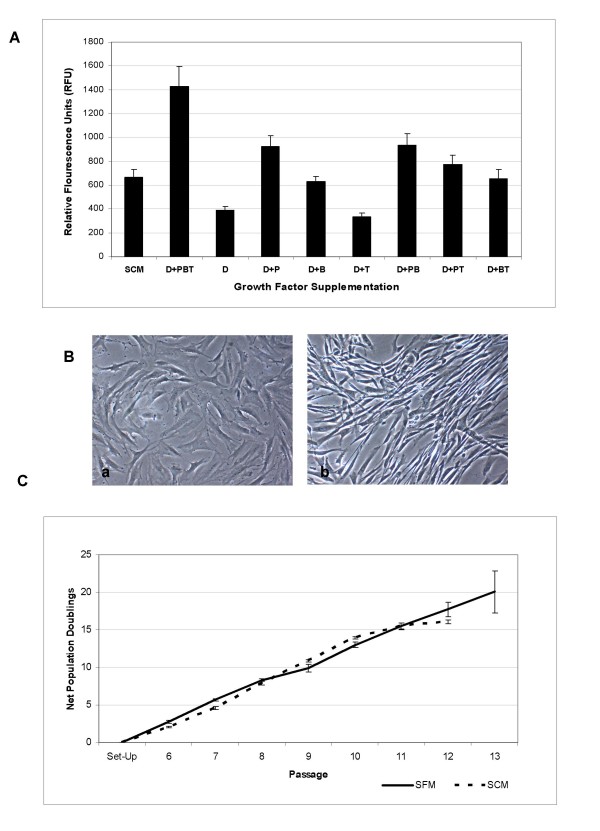
**Optimization of a serum-free growth medium**. **(a) **Fluorescence-based quantification of human MSC proliferation in deficient serum-free medium (SFM) supplemented with various combinations of recombinant human growth factors or in serum-containing medium (SCM) by using the CyQUANT NF Cell Proliferation Assay. D, growth factor-deficient SFM; P, SFM supplemented with recombinant human PDGF-BB; B, SFM supplemented with recombinant human bFGF; T, SFM supplemented with recombinant human TGF-β1. **(b) **Morphology of passage 4 human MSCs expanded in (a) SCM and (b) SFM. Magnification, 10× objective. **(c) **Recovered four-donor pooled human MSCs (passage 5) expanded under optimized conditions for eight additional passages (48 days) in SFM or SCM.

**Figure 2:**
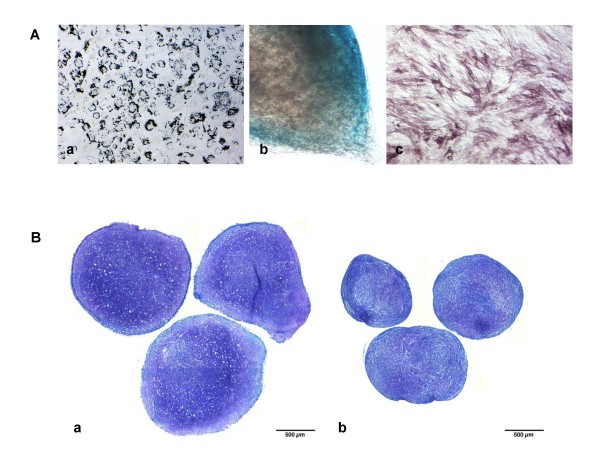
**Differentiation capacity of serum-free expanded human MSCs**. **(a) **Day 14 differentiation cultures from cells expanded for eight additional passages in SFM revealed trilineage mesoderm differentiation, as shown by positive (a) Oil Red O staining for adipocytes, (b) Alcian blue staining for chondrocytes, and (c) alkaline phosphatase staining for osteoblasts. (a, c) 10×, (b) 20× objective. **(b) **Toluidine blue staining of day 21 chondrogenic micromass-differentiation cultures after three passages in (a) serum-free medium or (b) serum-containing medium.

**Figure 3:**
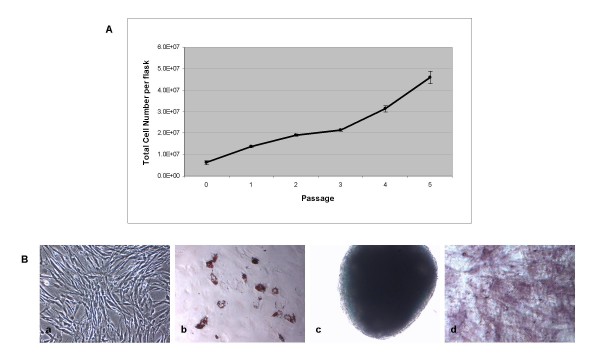
**Primary isolation, culture expansion, and differentiation of human bone marrow-derived MSCs**. **(a) **Net total cell expansion per flask for primary cultures grown in SFM for five continual passages. **(b) **(a) Morphology of passage 1 human bone marrow-derived MSCs in SFM. (b) Passage 5 MSCs in SFM were differentiated for 14 days and analyzed with (b) Oil Red O staining for adipocytes, (c) Alcian blue staining for chondrocytes, and (d) alkaline phosphatase staining for osteoblasts. a-d, 10× objective.

The developed serum-free supplement consisted of a blend of essential amino acids, inorganic salts, and other components, along with an optimized mix of the recombinant human growth factors PDGF-BB, bFGF, and TGF-β1. This blend was formulated based on the effect of each individual growth factor or combination of factors on human MSC proliferation. Although supplementation of certain individual factors or combinations of two factors provided enhanced expansion compared with a growth factor-deficient serum-free medium (SFM) or 10% fetal bovine serum-containing medium (SCM) (Figure 1a), it was the combination of all three growth factors together that provided a robust synergistic effect on human MSC proliferation. It is noteworthy that along with cell proliferation, a drastic change in cell morphology was observed in the presence of all three growth factors. Whereas growth factor-stimulated cells expanded in SFM displayed a small, spindle-shaped morphology, cells grown in SCM exhibited a more flattened, characteristic fibroblast-like morphology (Figure 1b). In addition, cells continually propagated in SFM tended to grow in distinct bundles of cells, as compared with cells in SCM, which grew as a more-flattened, uniform monolayer of cells. To test the ability of the optimized SFM to support continual propagation of multipotent MSCs, cryopreserved human bone marrow-derived MSCs from a passage 4, four-donor pool were used as an initial starting population. From this pool, cells were recovered in SCM and transitioned directly to SFM on CELLstart™ or human plasma fibronectin-coated vessels. Cells cultured in SFM without the presence of a substrate on tissue culture-treated flasks were unable to achieve sufficient initial adherence after seeding and did not proliferate in culture (data not shown). From this point, human MSCs were expanded in the SFM for eight continual passages (48 days; passage 13 total) (Figure 1c), after which cultures slowed and were therefore terminated. It is noteworthy that cells expanded in this SFM required a higher cell-seeding density (~1 × 10^4 ^cells/cm^2^) compared with that historically recommended for traditional SCM cultures [11]. As cells grown in SCM reached confluence at a lower density, the resultant optimal passaging frequency of cells seeded at the same density differed between SCM and SFM cultures and made a direct side-by-side comparison more difficult. To achieve such a side-by-side comparison, cells in SCM were seeded at a lower seeding density (~3 × 10^3 ^cells/cm^2^), and net population doubling was used as a measure to compare the culture systems. To test the multipotentiality of human marrow-derived MSCs expanded in SFM, a traditional trilineage mesoderm differentiation protocol was used. As shown in Figure 2a, human bone marrow-derived MSCs grown for eight passages in SFM retained the ability to differentiate into adipocytes, chondrocytes, and osteoblasts, as shown by Oil Red O, Alcian blue, and alkaline phosphatase staining, respectively. In addition, human bone marrow-derived MSCs expanded in SFM from a single donor, passage 1 bank revealed robust chondrogenesis after three passages in SFM, as shown by dark purple staining and the distinct morphologic presence of chondrocytes in micromass pellet cultures (Figure 2b). To explore the ability of SFM to support primary human bone marrow-derived MSC culture, fresh bone marrow mononuclear cells isolated by using a standard density-gradient centrifugation protocol were seeded into SFM and SCM. As shown in Figure 3b, after 8 days, primary MSCs (passage 0) in SFM displayed a distinct spindle-shaped morphology. In addition, MSCs isolated under serum-free conditions showed continued expansion potential with retained trilineage mesoderm differentiation potential (Figure 3a and 3b).

**Figure 4:**
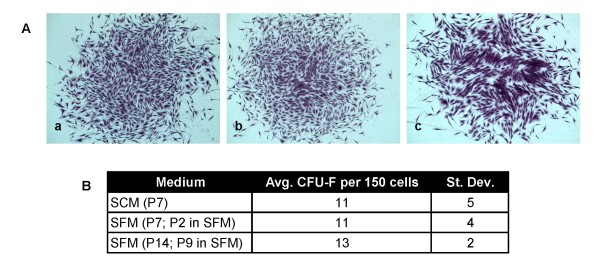
**CFU-F assay of serum-free expanded human MSCs**. **(a) **Representative images of 14-day CFU-F colonies initiated from (a) passage 2 SFM cultures (passage 7 total), (b) passage 2 SCM cultures (passage 7 total), and (c) passage 9 SFM cultures (passage 14 total). **(b) **Enumeration of CFU-F colonies (those ≥ 50 cells) at each respective passage. Data shown represent *n *= 5 technical replicates.

Historically, the colony-forming unit-fibroblast (CFU-F) assay has been used as a correlative assay to test the presence of clonal mesenchymal stem cells within a given cell population [2,12]. To test the potential of cells expanded in SCM versus SFM, human MSCs passaged under each respective condition were placed into a CFU-F assay. As shown in Figure 4, cells grown in SFM for two passages displayed a similar CFU-F potential compared with cells in SCM (11 ± 4 vs. 11 ± 5 (SD) respectively). In addition, cells continually propagated in SFM for nine passages displayed a similar CFU-F potential to that observed at passage 2.

To test further the phenotype of human MSCs expanded in SFM, cell-surface antigens were analyzed by flow cytometry (passage 3) (Table 1) and qRT-PCR (passage 4) (Table 2). Human MSCs expanded in SFM displayed a characteristic surface profile [13], including positive expression of CD73, CD90, and CD105, as well as negative expression of CD11b, CD14, CD19, CD34, CD45, and CD79a. Interestingly, unlike cells grown in SCM, cells expanded in SFM displayed a significantly higher level of the type VI intermediate filament Nestin compared with cells expanded in SCM (Table 2).

**Table 1:** Flow-cytometric analysis of expanded human MSCs

Flow cytometry	SFM	SCM
**target**	**% Positive (flow cytometry)**	

Negative human MSC markers		

CD11b	0.49	0.50
CD34	0.26	1.04
CD45	1.58	1.91
CD79a	0.57	0.90

Positive human MSC markers		

CD44	99.14	99.73
CD73	99.38	99.90
CD90	99.51	99.63
CD105	97.95	99.78

Analysis of known positive and negative human MSC surface antigens by flow cytometry for cells expanded in both SFM and SCM for three passages.

**Table 2:** Quantitative real-time PCR analysis of expanded human MSCs

qRT-PCR	SFM		SCM	
**Target**	**Expression**	**ΔCt**	**Expression level**	**ΔCt**

Positive Human MSC markers				

CD44	N/A	N/A	N/A	N/A
CD73	++++	2.55	++++	3.53
CD90	++++	2.40	++++	2.63
CD105	++++	2.63	++++	1.82
Nestin	+++	5.79	++	11.17

Quantitative real-time PCR analysis of known positive human MSC surface antigens for cells expanded in both SFM and SCM after four passages. ΔCt, Ct (gene-of-interest) - Ct (GAPDH), ++++, very high; +++, high; ++, moderate; +, low.

**Figure 5:**
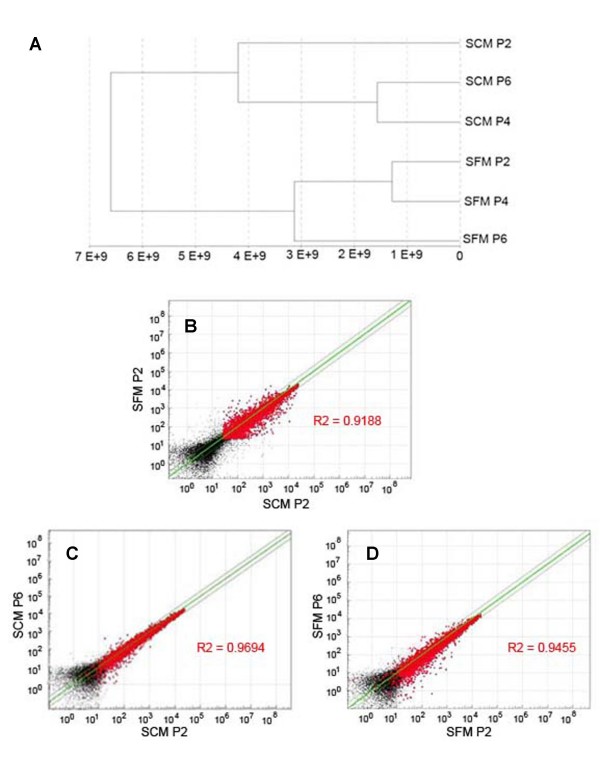
**Transcriptome comparison of MSCs cultured in serum-free and serum-containing media**. **(a) **Dendrogram showing the relatedness of cells based on global gene expression obtained by using Illumina bead array and analyzed by using Bead Studio. **(b-d) **Pairwise comparison of global gene-expression profiles of MSCs maintained in SCM and SFM. Data points >0.99 detection limit are represented in red. Points lying outside the green line represent genes with greater than twofold difference, whereas *R*^2 ^values closer to 1 suggest similarity in global gene expression. **(b) **Human MSCs in SCM and SFM after passage 2 (P2). **(c) **Comparison of cells grown for two passages (P2) versus cells grown for six passages (P6) in SCM. **(d) **Comparison of cells grown for two passages (P2) versus cells grown for six passages (P6) in SFM.

To identify genotypic differences between cells expanded under these two expansion-media conditions, a global gene-expression analysis was used. Gene-expression analysis of MSC samples cultured in SCM or SFM was carried out by using the Illumina bead array containing 46,000 full-length and splice-variant transcripts from the Human RefSeq database. Each transcript is represented by a 50-mer probe present on an average of 30 beads within the BeadArray that provides high selectively and reproducible results. The total number of genes detected over 0.99 confidence was considered for analysis. Samples were clustered to plot a dendrogram (Figure 5a). Dendrograms represent a form of hierarchical clustering, and are plotted with the raw global gene-expression data to determine the relatedness of samples. Clustering of the samples represents the euclidean distance, with clustering together showing greater similarity than those far from each other. Cells grown in SFM for varying passages clustered closer to each other than cells grown in SCM. Further pairwise comparison by using scatterplots was carried out to determine the correlation between the global expression of cells grown for varying periods in SCM and SFM. This allows estimation of variability of the two samples based on the correlation coefficient (R^2^) value, with a value of closer to 1 indicating relatedness and a value farther from 1 indicating variability. Figure 5b shows a pairwise comparison of the global gene-expression profile of human MSCs expanded in SFM versus SCM. Despite the clustering of SCM cells away from SFM in the dendrogram, the R^2 ^value determined by scatterplot is 0.9188, and hence, they are not drastically different from each other. Further continual propagation of MSCs in SFM medium shows minor changes in gene-expression profiles, and the extent of this change is similar to that observed with MSCs continually grown in traditional SCM. Further, cells continually propagated in either formulation for an additional four passages (Figure 5c and 5d) show close correlation with starting cultures, indicating that the cells do not have a significant deviation in gene expression as a result of continual propagation in either expansion medium. The complete gene-expression data are represented in Supplementary Table 1 in Additional file 1.

## Discussion

To facilitate the transition of human MSC biology from basic studies to clinical application, advances in the reagents used to expand these therapeutically relevant cells has become an absolute necessity. One of the most important achievements in this area will be the transition from SCM to a better-defined SFM. Although recent efforts have shown that human placenta and bone marrow-derived MSCs can be isolated and expanded over the short term in serum-free medium [14], no published work had shown the ability to expand human MSCs under serum-free conditions for the long term in culture. To achieve such a feat, the optimized serum-free medium StemPro^® ^MSC SFM was developed [10]. Perhaps one of the most important features of this serum-free formulation was the optimization of the growth factors required for optimal human MSC expansion. Three of the most important growth factors described as playing a role in both human MSC proliferation and differentiation are PDGF-BB, bFGF, and TGF-β1 [10]. Whereas PDGF-BB and bFGF had positive effects on cell growth individually, TGF-β1 appeared to provide no enhancement of cell proliferation on its own. Interestingly, although combinations of any two growth factors appeared to provide minimal or no significant enhancement of cell proliferation compared with single factors, the combination of all three factors provided an obvious synergistic effect.

To test the ability of the optimized SFM to support continual propagation, established MSC cultures in SCM were transitioned directly into SFM. It is important to note the difference in morphology of cells in SFM versus SCM. On removing from serum, cells grown in SFM adopted a more spindle-shaped morphology with a distinct cell-growth pattern. This morphology allowed cells in SFM to be grown at much higher densities compared with cells grown in SCM. As a result, this morphologic change caused cells cultured in SCM versus SFM to require different seeding densities or passage frequencies or both. Also, unlike cells grown in SCM, which can be grown optimally at very low seeding densities, cells in SFM appeared to perform better at higher seeding densities (~1 × 10^4 ^cells/cm^2^), facilitating optimal cell expansion and continual propagation. The ability to expand human MSCs at high densities with retained multipotency provides a beneficial approach.

As the MSC field moves toward the establishment of more-robust and efficient clinical protocols, the ability to expand large numbers of cells at high density quickly by using fewer culture wares and reagents may prove very useful. It is noteworthy that cells transitioned directly from SCM to SFM may have shown enhanced proliferation for the first one to two passages directly out of SCM. After two passages, SFM cultures provided an average expansion rate (doubling time; average for eight passages) similar to that achieved with SCM (46.3 ± 7.1 versus 52.5 ± 6.9 (SD) hours, respectively). In addition, similar to that described recently [15], our optimized SFM was capable of supporting expansion of MSCs directly from primary human bone marrow. Unlike typical observations for primary cultures in SCM, those in SFM did not result in fibroblast colonies, but rather generated somewhat evenly distributed proliferative MSC-like cells throughout the culture flask. The reason for this different growth pattern is unknown, but may be a result of the substrate (human plasma fibronectin or CELLstart™) required for MSC growth in SFM or the presence of recombinant human growth factors (PDGF-BB, bFGF, and TGF-α1), which have been described as having a migratory effect on MSCs [16-19].

It is important to note that the described multipassage expansion experiments were conducted by using (a) pooled passage 4 human MSCs isolated in SCM from four individual donors and transitioned to SFM; (b) single-donor human MSCs isolated in SCM and transitioned to SFM; and (c) primary human MSCs isolated directly in SFM from a single donor. Although the presented data do not directly address donor-to-donor variability and the efficacy for human MSCs isolated in SCM versus SFM, it is noteworthy that Agata *et al*. [15] directly addressed these questions. In this work, the authors concluded that the efficacy of primary cultures was greater in SFM compared with SCM and that this effect was independent of specific donors.

The use of pooled human MSCs in our study was intended to overcome donor-to-donor variability and provide a reliable and consistent population of MSCs for experimentation. Although this approach certainly provides a consistent average population of cells for relatively short-term cultures, it is possible during long-term culture that human MSCs from a single donor (or donors) with faster growth rates may overtake the cultures and therefore provide a less-average and more-optimal result.

Despite the nontraditional growth patterns, morphology, and expansion protocols required for growing human MSCs in SFM, expanded MSCs in this medium still displayed multipotentiality with successful differentiation to adipocytes, osteoblasts, and chondrocytes. As the chondrogenic potential of cultured MSCs is often the first differentiation capacity lost during *in vitro *expansion (unpublished results), the apparent retained robust chondrogenic differentiation potential shown through pellet cultures and Toludine Blue O staining is a promising characteristic provided by this serum-free medium. As described by Agata *et al*. [15], human bone marrow-derived MSCs isolated and expanded in SFM still retained the competence for osteogenic induction and ectopic bone formation.

As proposed previously by the International Society for Cell Therapy [13], a population of multipotent human MSCs must possess a specific cell-surface antigen expression profile. In accordance with that defined by this group, cells grown in SFM displayed positive expression of CD73, CD90, and CD105 and negative expression of CD11b, CD14, CD19, CD34, CD45, and CD79a, as detected by quantitative real-time PCR and flow cytometry. Interestingly, cells grown in SFM displayed significantly enhanced expression of the intermediate filament nestin. Whereas nestin was originally identified as a marker for neural stem cells [20], it has since been reported to be present in numerous cell types including human MSCs [21]. As pericytes have been suggested the *in vivo *origin of *in vitro *expanded human MSCs [22,23] and have been reported to express nestin *in vivo *[24,25], it may be possible that nestin expression represents a more *in vivo *phenotype for expanded MSCs.

After an analysis of gene-expression data generated on SFM versus SCM cultures, dendrogram results reveal that, over multiple passages, cells grown in SFM clustered together and cells in SCM cluster together. Despite this clustering, the pairwise comparison of SFM versus SFM global gene expression (passage 2; *R*^2 ^= 0.9188) suggests a close correlation between the expression pattern of MSCs grown in SCM and SFM. In addition, cells grown in either medium for additional passages continue to show close correlation with earlier passaged cells in the same medium, suggesting minimal genotypic changes. Although differences in gene-expression analysis between MSCs grown in SFM and SCM do not seem to have an impact on either proliferation or multipotency, a more-detailed analysis on the effect these differentially expressed genes may play on overall MSC biology must be further explored. It is important to note that the bulk of the characterization data provided in this work was conducted on cells isolated by using SCM and thereafter transitioned to SFM and further expanded under such conditions. As the possibility exists that the standard SCM isolation procedure may result in the selection of a certain population of primary cells (MSCs) with a distinct phenotype, it cannot be ruled out that such characterization experiments (that is, gene-array studies) may differ when comparing cells isolated under SCM versus SFM conditions.

In addition to the data presented here focusing on the expansion of human bone marrow-derived MSCs, it has also been observed that the described SFM may be used for expansion of human MSCs from other sources including umbilical cord-derived MSCs and adipose-derived stem cells (ADSCs) (unpublished data). Despite the preliminary data suggesting the ability to expand these additional MSC populations in SFM, future studies will be necessary to determine whether additional supplementation is necessary most efficiently to expand these populations.

## Conclusions

The development of a serum-free medium for the isolation and expansion of human MSCs represents a necessary step in developing the tools required to study human MSCs in a consistent and reproducible manner. By eliminating the use of fetal bovine serum, an inherently variable reagent, this novel serum-free medium will provide the ability for researchers from different laboratories to conduct studies with similar reagents, fostering scientific advances and discovery. Future studies will be necessary to realize completely the effect this medium has on MSC biology *in vivo*. Finally, as this described SFM contains nonhuman animal-origin materials, future optimization and medium development leading to a serum-free, xenogeneic-free and therefore a more clinically relevant culture system will be necessary.

## Abbreviations

bFGF: basic fibroblast growth factor; CFU-F: colony-forming unit fibroblast; DMSO: dimethyl sulfoxide; DPBS: Dulbecco's phosphate-buffered saline; FBS: fetal bovine serum; MSC: mesenchymal stem cell; PDGF-BB: platelet-derived growth factor-BB; qRT-PCR: real-time quantitative PCR; SCM: serum-containing medium; SFM: serum-free medium; TGF-β1: transforming growth factor, β1.

## Competing interests

LC, UL, MR, and MV declare financial competing interests, as all are employees of Life Technologies. Life Technologies markets reagents and tools for stem cell biology. LS declares no financial competing interests.

## Authors' contributions

LS performed MSC chondrogenic differentiation experiments. UL performed gene array analysis, manuscript drafting, and editing. LC conceived the experiments, performed all remaining experiments, manuscript drafting, and editing. MR and MV participated in conceiving and coordinating the studies and edited the manuscript.

## Supplementary Material

Additional file 1 **Supplemental Table 1: SCM vs. SFM Array Data**. Complete gene-expression data set (passages 2, 4, and 6) for human MSCs expanded in SCM and SFM. Click here for file
